# Computed Tomography Analysis of Postsurgery Femoral Component Rotation Based on a Force Sensing Device Method versus Hypothetical Rotational Alignment Based on Anatomical Landmark Methods: A Pilot Study

**DOI:** 10.1155/2016/4961846

**Published:** 2016-01-04

**Authors:** Stefan W. Kreuzer, Amir Pourmoghaddam, Kevin J. Leffers, Clint W. Johnson, Marius Dettmer

**Affiliations:** ^1^Memorial Bone & Joint Research Foundation, 1140 Business Center Drive, Suite 101, Houston, TX 77043, USA; ^2^Department of Orthopaedic Surgery and Rehabilitation, The University of Texas Medical Branch, 301 University Boulevard, Galveston, TX 77555, USA; ^3^UT Physicians Orthopedics, 9305 Pinecroft Drive, Suite 400, The Woodlands, TX 77380, USA; ^4^Research Focus Cognition Sciences, Division of Training and Movement Sciences, Faculty of Human Sciences, University of Potsdam, Am Neuen Palais 10, Building 12, 14469 Potsdam, Germany

## Abstract

Rotation of the femoral component is an important aspect of knee arthroplasty, due to its effects on postsurgery knee kinematics and associated functional outcomes. It is still debated which method for establishing rotational alignment is preferable in orthopedic surgery. We compared force sensing based femoral component rotation with traditional anatomic landmark methods to investigate which method is more accurate in terms of alignment to the true transepicondylar axis. Thirty-one patients underwent computer-navigated total knee arthroplasty for osteoarthritis with femoral rotation established via a force sensor. During surgery, three alternative hypothetical femoral rotational alignments were assessed, based on transepicondylar axis, anterior-posterior axis, or the utilization of a posterior condyles referencing jig. Postoperative computed tomography scans were obtained to investigate rotation characteristics. Significant differences in rotation characteristics were found between rotation according to DKB and other methods (*P* < 0.05). Soft tissue balancing resulted in smaller deviation from anatomical epicondylar axis than any other method. 77% of operated knees were within a range of ±3° of rotation. Only between 48% and 52% of knees would have been rotated appropriately using the other methods. The current results indicate that force sensors may be valuable for establishing correct femoral rotation.

## 1. Introduction

Total knee arthroplasty (TKA) is a challenging, yet successful, intervention for patients with knee osteoarthritis [1]. However, despite its success in relieving pain, advances in technology, and improved surgical techniques, 11%–19% of patients are unsatisfied with the outcome of this intervention [2–5].

Traditionally, TKA is initiated with bone cutting to establish a neutral mechanical axis followed by (often extensive) necessary soft tissue releases to balance and match the flexion/extension gaps with the distal femoral and proximal tibial resections at right angles to the mechanical axis. Although computer-assisted surgery has made the TKA more reproducible and predictable, resulting in more accurate and precise bone alignment [6], accuracy of soft tissue balancing continues to be a challenge. This is due to a number of involved systems contributing to stability and proper function of the knee joint. These are (1) bony structures of the knee, (2) the static (or passive) stabilizers of the joint (medial and lateral collateral ligaments, capsule, and anterior and posterior cruciate ligaments), and (3) the dynamic (or active) stabilizers consisting of muscle-tendon units around the knee. An optimized balance among these systems is crucial to the successful outcome of TKA [7, 8].

Although bone alignment is critical, soft tissue balance has been shown to be just as important for physiologic functioning of the knee joint [7]. Subclinical instability can lead to a quadriceps avoidance gait and decreased ability regarding functions such as moving laterally, turning, cutting, carrying loads, and kneeling [3]. The importance of correct femoral rotation has been well documented due to its altering effect on patella alignment and flexion instability [9], range of motion, or polyethylene wear [10]. The latter may be increased in cases of incorrect rotation of the components, improper ligament balancing, inaccurate bone cuts, or prosthetic design [1, 11–13].

**Figure 1:**
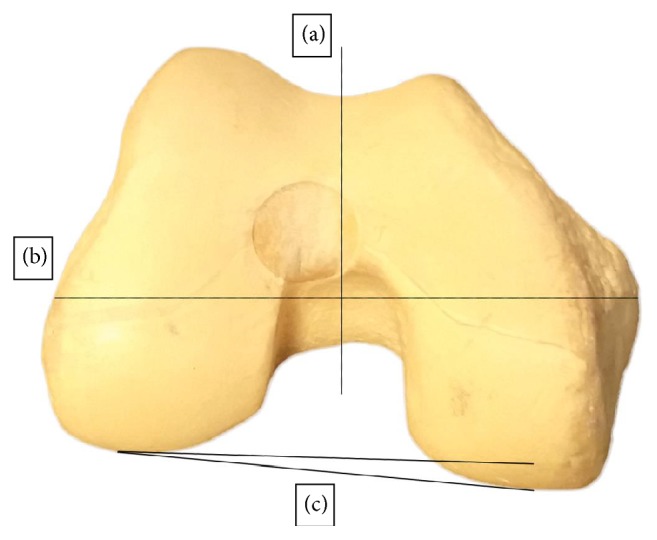
After registration of bony landmarks, that is, most anterior trochlear groove, most distal trochlear groove, medial and lateral epicondyle, and most posterior medial and most posterior lateral point via a computer-assisted navigation system, establishing femoral rotation can be based on 90° angle from anterior-posterior midline or “Whiteside's line” (a) or be based on anatomical transepicondylar axis (b). The conventional method uses the posterior condyles as a reference and prescribes three degrees' rotation (c).

There are several widely practiced methods to establish femoral component rotation. The more prominent ones are a conventional method of referencing to the posterior condylar axis with a standard external rotation of 3° (PCR), anterior-posterior line or “Whiteside's line” (AP-axis), transepicondylar axis (TEA) (Figure 1), and the gap balancing technique [14–19]; however, it is not yet clear which method is superior for femoral rotational component alignment [20].

Earlier research suggests that anatomic referencing methods carry at least a 10% chance of flexion gap imbalance, defined as greater than 3° of asymmetry from a balanced flexion space [21]. This may be due to interobserver differences and potential inaccuracy in registering/locating the specific bony landmarks or interindividual differences, for example, regarding the shape and proportions of the posterior condyles or anatomy of other knee structures.

Flexion gap asymmetry is believed to be the root cause of flexion instability, which can present as recurrent effusions, multiple areas of soft tissue tenderness, tibial translation, and instability without giving way [22–25]. Although infection is the most common cause of failure, instability has been identified as a common issue too [26, 27]. Opportunity for nonoperative treatment is limited in those cases [28], and there still are revisions required in a number of cases [29].

In an effort to address this issue, an alternative evaluation instrument, the eLIBRA Soft Tissue Force Sensor (Synvasive Technology, Reno, NV), was developed. The tool is designed to provide a more objective measurement option for the forces occurring within the medial and lateral compartments prior to implant placement. The device allows for dynamic knee balancing (DKB), which describes real-time assessment of the compressive forces within the medial and lateral compartment, which provides surgeons with an opportunity to dynamically and instantly modify internal/external rotation of the femoral component [30, 31], while accounting for the varus/valgus angle of the tibial cut.

DKB dictates femoral component rotation on the basis of ligament balance and force measures rather than the existing methods of referencing femoral rotation based on anatomical landmarks. Considering the risk of an asymmetric flexion space associated with the use of traditional methods [21], DKB has become more prominent in TKA surgeries. While retaining ligament balance in TKA, it is possible that this technique also leads to higher precision of rotational alignment to the anatomical axis. The technique has been shown to be effective regarding accurate femoral rotation and associated patient-reported outcomes [31]; however, there is lack of scientific evidence concerning potential anatomical rotational alignment advantages over other techniques. The primary objective of this study was to compare efficiency of DKB versus other methods for rotational implant alignment as evaluated by postsurgery computed tomography (CT). We hypothesized that femoral rotation based on DKB would be superior to the anatomical landmark methods in terms of deviation from the anatomical TEA (aTEA) as located via postsurgery CT.

## 2. Materials and Methods

### 2.1. Patients

The experimental protocol was approved by the Western Institutional Review Board and all patients signed informed consent before the start of the study. Over the course of seven months, 31 patients (20 females and 11 males) with a diagnosis of osteoarthritis underwent TKA by using OrthoMap ASM Knee Navigation (Stryker Co., Kalamazoo, MI). All patients received a Stryker Triathlon Knee (Stryker Co., Kalamazoo, MI). Average age of patients was 69.6 ± 8.6 years, height was 167.6 ± 10.9, and body mass index (BMI) was 33.2 ± 6.7 kg/m^2^. Inclusion criteria were varus deformity of less than 15 degrees and valgus deformity less than 20 degrees (measured during surgery via navigation). Patients were excluded if there had been earlier distal femoral or proximal tibial osteotomies or if there were any deformities due to malunion of either femur and/or tibia after fracture or if there were more than 3 degrees of varus tibial plateau. Revision patients were excluded as well. Such exclusion criteria were established since they could have contributed to a soft tissue envelope that could be contracted or scarred and therefore could have influenced results in this study.

All knees underwent a median parapatellar incision. The surgical technique included a varus tibial cut to approximate the joint line and to generate a more varus alignment of the tibial component [32]. To avoid early failure, tibial varus/valgus was maintained within ±3 degrees. Valgus and neutral tibial plateaus were resected in a neutral manner (a tibial varus resection up to 3 degrees was performed for varus tibial plateaus, which is associated with a more anatomic alignment of the joint surface and better function [33]). With regard to preoperative alignment, 27 knees were in varus, three were in valgus, and one was neutral. 75 percent of the distal femoral cuts were from 0° to 2° of neutral (range: 1° valgus to 3° varus), and 87% of the proximal tibial cuts were from 0° to 2° (range: 1° valgus to 3° varus). There were no complications and/or revisions.

### 2.2. Procedure

**Figure 2:**
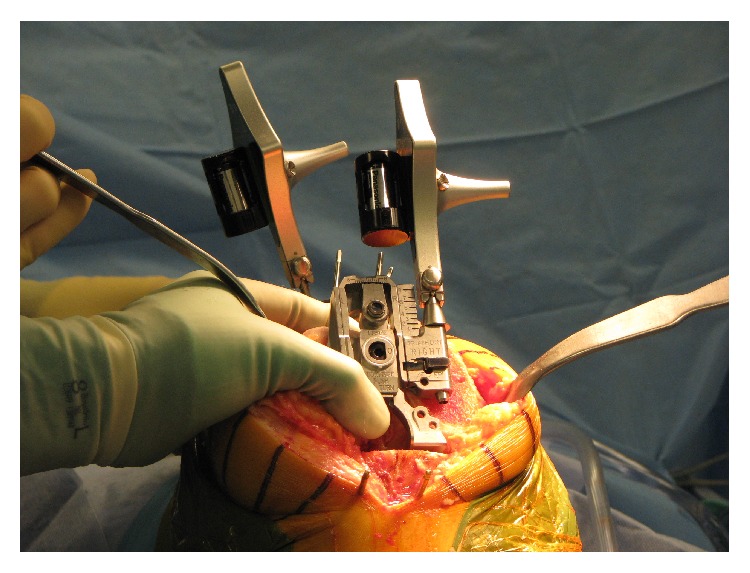
Stryker Triathlon reference jig used for comparison of landmark methods, force sensor technique, and posterior condyles referencing.

After a femoral tracker was placed inside the incision and a tibial tracker was placed through separate small incisions, the routine registration process was completed (e.g., condyles, tibial plateau, and epicondyles) according to a protocol prescribed by the navigation technology. Before any bone cuts were made, limb alignment and range of motion were measured and recorded using the OrthoMap software. Subsequently, the surgeon proceeded with the bone cuts, starting with the femoral cut and then the tibial cut. The angles of the femoral cut and the tibial cut were recorded prior to balancing the extension gap. A posterior condyles-referenced Triathlon femoral rotation guide was attached and set to 3° of external rotation. This position was used as a reference point and to investigate differences between posterior condyles referencing and the other methods (Figure 2).

**Figure 3:**
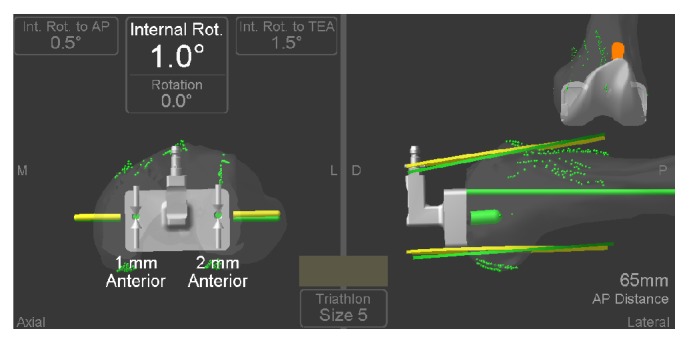
Stryker navigation results including internal rotation according to AP and TEA and average of the two.

The Stryker navigation system was then used to obtain several measurements: current rotation (as prescribed by the posterior condyles reference guide) relative to the previously registered landmarks and associated TEA and AP-axis/Whiteside's line (Figure 3). This data was used to calculate the theoretical placement of the implant according to each specific method.

**Figure 4:**
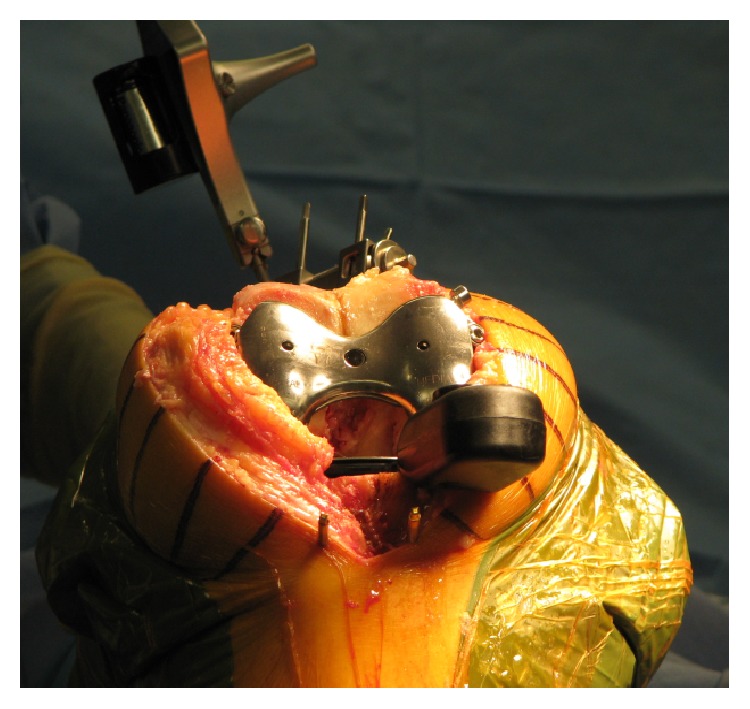
eLIBRA force sensor device for soft tissue balancing during TKA. Compressive forces are measured (separately at each condyle) in flexed knee position to create a balanced gap.

Next, the eLIBRA femoral component was placed on the distal femur by using two screws. The appropriate tibial insert thickness with soft tissue force sensor was inserted in order to achieve medial compartment stability and soft tissue tone (Figure 4).

The patella was then reduced and the knee flexed to 90° while a wrench was used to adjust femoral rotation until equal force was distributed across both the medial and lateral compartments to create a balanced soft tissue envelope in flexion.

Femoral rotation using the DKB (in relation to AP, TEA, and PCR) was then recorded by employing a technique similar to the method described earlier, and using the OrthoMap system. Femoral rotation holes were drilled through the eLIBRA femoral component to direct the Triathlon 4-in-1 cutting block. After the cutting process was completed, the arthroplasty components were implanted based on the DKB system's prescription. The patella was also resurfaced using the conventional instruments of the triathlon knee system. The procedure was completed by closing the arthrotomy and skin.

### 2.3. Data Processing

**Figure 5:**
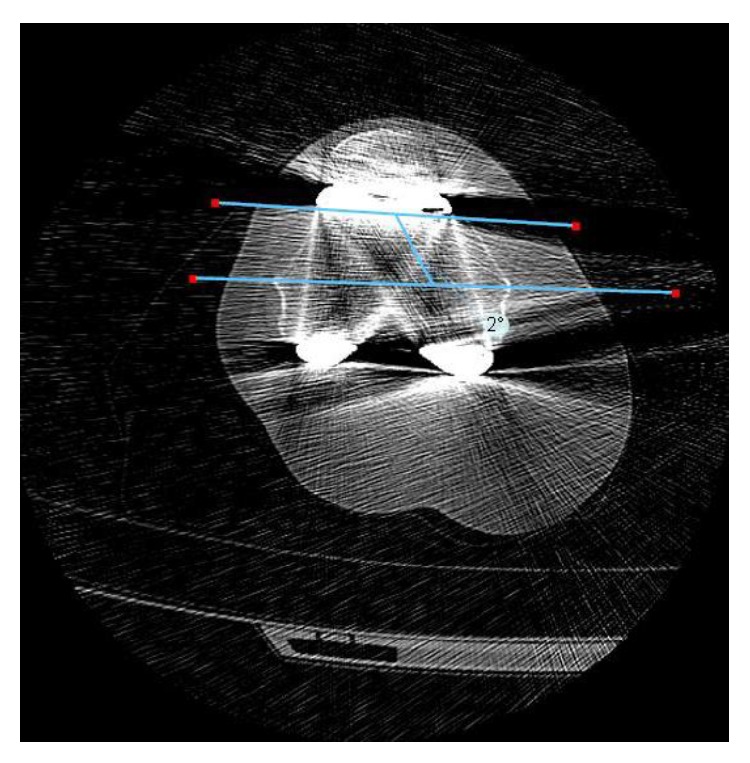
CT-scan on operative knee (after surgery). Measurement of femoral rotation in regard to anatomical epicondylar axis performed by two independent observers.

Postoperative CT-scans with 2 mm intervals were obtained on the operative knee in 31 individuals. Measurements of the femoral component rotation in relation to the anatomic TEA were recorded independently by two investigators using TraumaCad software (TraumaCad 2.2, Voyant Health, Columbia, MD). The lateral epicondyle was identified and used as a rotational axis when scrolling through the axial images to find the anatomic medial epicondyle. Once the anatomical TEA (aTEA) was identified, the femoral implant line and anterior cut were identified. The computerized angle tool was then used to measure the angle between the femoral component and the aTEA (Figure 5), a technique similar to that used by Witoolkollachit and Seubchompoo [34].

All angular data obtained this way consisted of either positive (external rotation) or negative values (internal rotation). The CT data and data from intraoperative measurements then were used to calculate the theoretical placement of the femoral components according to the respective anatomical landmark methods (TEA, AP, and PCR). Therefore, the intraoperative theoretical positioning according to other methods was compared to the actual positioning according to DKB in relation to the CT-scan.

### 2.4. Statistical Analysis

CT results from two independently conducted measures (two observers) were compared using *t*-tests to determine if there were interobserver differences. Absolute values for deviation from aTEA for each method were then analyzed using repeated measures ANOVA. Additional Holm-Bonferroni-corrected tests were employed for pairwise comparisons.

Cochran's *Q* test was utilized to investigate the proportionality of distribution of cases that were either within or in excess of ±3° or ±5° rotation. McNemar's tests were then applied (Holm-Bonferroni adjusted for multiple comparisons) to further test specific differences between the DKB method and the other techniques.

## 3. Results

### 3.1. Computed Tomography Data: Deviation from aTEA

**Figure 6:**
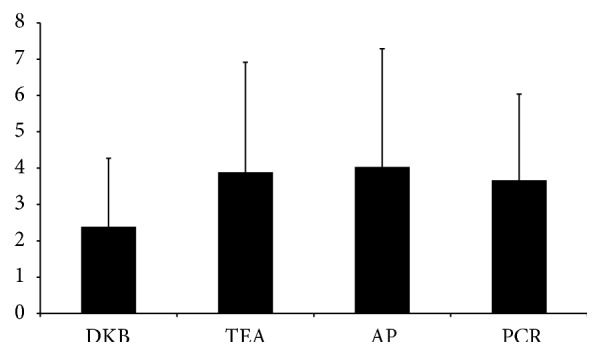
Mean deviations (in degrees) and standard errors obtained from CT-determined aTEA for each method.

No significant difference was found between the CT results obtained by the two independent observers (*P* = 0.7). There was a significant main effect of employed rotation method on absolute deviation from the aTEA, *F*(2.463,73.9) = 3.580, *P* = 0.03. Further pairwise comparisons revealed significant differences between the DKB method and TEA method (*P* = 0.02), between DKB and AP method (*P* = 0.04), and between DKB and PCR method (*P* = 0.02). The DKB method showed the lowest rotational deviation from CT-determined aTEA (Figure 6).

### 3.2. Computed Tomography Data: Number of Cases within Range of ±3° and ±5°

The four methods were then compared to one another to determine what percentages were within 3° and 5° of rotation to the aTEA. The DKB method placed the femoral component within 3° of the epicondylar axis in 77% of the observed cases. Likewise, the PCR, TEA, and AP rotations were within 3° of the epicondylar axis 48%, 48%, and 52%, respectively (Table 1). Cochran's *Q* indicated a difference between the four proportions, *χ* ^2^(3, *n* = 31) = 9.00, *P* = 0.03. Subsequent McNemar's tests indicated significant differences between DKB and TEA and analysis revealed a significant proportional difference between DKB and PCR method (*P* = 0.01), between DKB and TEA (*P* = 0.02), and between DKB and AP (*P* = 0.04).

Analysis of proportion of cases within ±5° of rotation to aTEA showed that DKB yielded 90% success. Femoral rotations were within that specific boundary in 77% (PCR), 71% (TEA), and 61% (AP) of the cases (Table 2). Cochran's *Q* test indicated a significant difference between techniques, *χ* ^2^(3, *n* = 31) = 10.222, *P* = 0.02. Subsequent analysis revealed significant differences between DKB and AP (*P* < 0.001) and between DKB and TEA (*P* = 0.02). There were no differences between DKB and PCR method (*P* = 0.21).

## 4. Discussion

Flexion and extension balancing have been identified as a major contributor to the long-term success of TKA and patient satisfaction [21]. Adequate balancing is associated with the elimination of a number of postoperative issues that can lead to recurrent joint pain and lower than expected clinical outcomes [22]. Insufficient soft tissue balancing can result in limitation to range of motion, malalignment, knee instability, maltracking patella, premature mechanical failure, and pain [10, 35]. Many TKA systems today offer various methods to achieve correct bone alignment through intramedullary/extramedullary guides, patient-specific cutting blocks, or 2D/3D computer navigation, with varying efficiency [36–38]. None of the systems offers a standardized, optimal way to measure soft tissue balance. Spacer blocks, lamina spreaders, and tensioning devices have all been used in gap balancing techniques [39, 40]. The purpose of this study was to investigate the differences in postsurgery rotational alignment between several methods, based on reliable and objective method (CT imaging).

In our study, the initial hypothesis was confirmed; the soft tissue envelope-based DKB method provided superior results in comparison to all other techniques. DKB aligned femoral rotation more accurately in a larger number of cases. This is remarkable, since the data shows that not only does the DKB method (by definition) balance the flexion gap based on soft tissue features as an orientation, but it may be a better navigational tool for rotational alignment relative to the aTEA in comparison to other methods.

One potential explanation is the aforementioned occurrence of navigation error during the initial registration process [41], issues concerning the technique of establishing axes [42], residual cartilage [43], or the problem of interindividual differences regarding anatomy [44, 45]. DKB is independent of the registration process, which diminishes influence of registration inaccuracy or individual factors.

**Figure 7:**
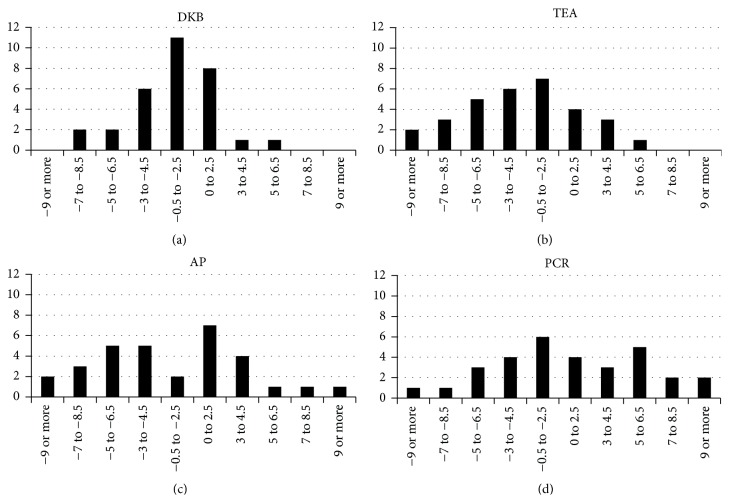
Histograms of rotational alignment (in degrees) based on DKB and alternative methods.

Analysis of proportion of case frequencies (cases within ±3° or ±5° of rotation) revealed that there were significant differences between the techniques related to their effectiveness in achieving low error ranges. The DKB technique leads to more cases within 3 degrees of rotation than the hypothetical use of the alternative methods (Figure 7). For a range of ±5°, DKB was superior in comparison to AP and TEA method, but not to the PCR method. Our results are far less pronounced than those reported in an earlier study, where it was found that only 17.3% of axes were less than 5° from the TEA when 11 orthopedic surgeons utilized five alignment techniques (including one computer-assisted technique and four traditional techniques) to establish femoral rotational alignment [46]. Arguably, there has been consistent technological progress regarding surgical navigation since the publication of the earlier study, so the results presented here may to some extent be a reflection of improved technique and technology. Additionally, only a single, very experienced surgeon performed the surgeries included in this study; therefore we eliminated potential interindividual differences.

In our study, the DKB method showed even more accurate anatomic placement compared with previous studies by Witoolkollachit and Seubchompoo, who reported 47.5% of 40 mobile bearing TKAs within 3° of the anatomic TEA [34]. More research is needed to better understand the complex, interacting effects of applied methods on alignment characteristics. It has been suggested that a combination of kinematic and anatomic techniques may be valuable for accurate femoral rotational alignment [47] and for reduction of patellar maltracking [48], but there is no consensus yet whether the combined methods are superior to alternative techniques, such as the DKB system. Also, there is a need to evaluate the relationship between rotational alignment accuracy and survey-based outcomes, since alignment is a predictor that may be confounded by other intrinsic patient factors [49]. Therefore, it is not yet clear what effects the alignments according to specific methods have in the longer-term.

Additionally, the reduction of the patellofemoral joint has been shown to affect the pressure across the tibiofemoral joint [50, 51]. While using the DKB technology, we did find that the amount of force detected by the sensor differed depending on whether or not the patella was reduced or everted. The ability to assess patellofemoral tracking while including the extensor mechanism in the soft tissue balancing process is unique to this system and warrants further investigation.

A limitation of the current study is that all femoral components were placed using only the DKB method. The femoral rotations of the other methods were calculated on the basis of the presumption that the relation between the (hypothetical) rotations would remain constant. Hence, the limitation of the study is the lack of data from actual postsurgery CTs obtained after surgery using any of the alternative methods. Arguably, considering potential factors adding to the variability of each method regarding rotational alignment accuracy [21], there may be slightly different results regarding the deviation from the true TEA using such methods; however, this is beyond the scope of this study but would be an important and valuable next step to validate our findings on a larger scale, that is, in a future randomized-controlled study.

Another limitation is that balancing the flexion gap using the presented DKB technique requires integrity of the soft tissues involved. An incompetent MCL could result in errors in femoral rotation. In the current study, there was integrity of those structures in all patients included. A final limitation is the fact that all intrasurgical registration of bony landmarks was conducted by a single surgeon, whereas arguably there may be interrater differences regarding the accuracy of correct digitizing of structures, for example, epicondyles.

## 5. Conclusions

Achieving a balanced soft tissue envelope with anatomic femoral component rotation in TKA is crucial for replication of normal knee kinematics. Because of the numerous techniques, surgeon subjectivity, and patient variability, this objective is difficult to achieve and may be challenging to consistently reproduce. We present a novel approach to comparing an alternative method to current anatomical landmark methods. Using computer navigation with the DKB method showed promising results in our study regarding femoral rotation accuracy. The DKB technique may be a valuable tool in knee arthroplasty surgery, since it can be integrated seamlessly into a number of TKA surgical systems and is quick and easy to use.

## Figures and Tables

**Table 1:** Anatomic alignment (±3°). Femoral component rotation relative to aTEA measured by CT using DKB method, PCR, TEA, and AP anatomic methods.

	DKB	PCR	TEA	AP
±3°	24/31 (77%)	15/31 (48%)	15/31 (48%)	16/31 (52%)
>3°	7/31 (23%)	16/31 (52%)	16/31 (52%)	15/31 (48%)

**Table 2:** Anatomic alignment (±5°): femoral component rotation relative to aTEA measured by CT using DKB method, PCR, TEA, and AP anatomic methods.

	DKB	PCR	TEA	AP
±5°	28/31 (90%)	24/31 (77%)	22/31 (71%)	19/31 (61%)
>5°	3/31 (10%)	7/31 (23%)	9/31 (29%)	12/31 (39%)
